# Meconium obstruction in absence of cystic fibrosis in low birth weight infants: an emerging challenge from increasing survival

**DOI:** 10.1186/1824-7288-37-55

**Published:** 2011-11-14

**Authors:** Valentina Filomena Paradiso, Vito Briganti, Lucia Oriolo, Riccardo Coletta, Alessandro Calisti

**Affiliations:** 1 Università Cattolica Sacro Cuore, Scuola di Specializzazione in Chirurgia Pediatrica, Roma, Italy; 2 Azienda Ospedaliera San Camillo Forlanini, UOD Chirurgia ed Urologia Pediatrica, Roma, Italy

## Abstract

**Background:**

Meconium abnormalities are characterized by a wide spectrum of severity, from the meconium plug syndrome to the complicated meconium ileus associated with cystic fibrosis. Meconium Related Ileus in absence of Cystic Fibrosis includes a combination of highly viscid meconium and poor intestinal motility, low grade obstruction, benign systemic and abdominal examination, distended loops without air fluid levels. Associated risk factors are severe prematurity and low birth weight, Caesarean delivery, Maternal MgSO4 therapy, maternal diabetes. In the last 20 yrs a new specific type of these meconium related obstructions has been described in premature neonates with low birth weight. Its incidence has shown to increase while its management continues to be challenging and controversial for the risk of complicated obstruction and perforation.

**Materials and methods:**

Among 55 newborns admitted between 1992-2008 with Meconium Related Ileus as final diagnosis, data about Low Birth Weight infants (LBW < 1500 g) were extracted and compared to those of patients ≥ 1500 g. Hischsprung's Diseases and Cystic Fibrosis were excluded by rectal biopsy and genetic probe before discharge. A softening enema with Gastrografin was the first option whenever overt perforation was not present. Temporary stoma or trans appendiceal bowel irrigation were elected after unsuccessful enema while prompt surgical exploration was performed in perforated cases. NEC was excluded in all operated cases. Data collected were perinatal history and neonatal clinical data, radiological signs, clinical course and complications, management and outcome.

**Results:**

30 cases with BW ≥ 1500 g had an M/F ratio16/14, Mean B.W. 3052 g, Mean G.A. 37 w Caesarean section rate 40%. There were 10 meconium plug syndrome, 4 small left colon syndromes, and 16 meconium ileus without Cystic Fibrosis. Five cases were born at our institution (inborn) versus 25 referred after a mean of 2, 4 Days (1-7) after birth in another Hospital (outborn). They were managed, after a Gastrografin enema with 90% success rate, by 1 temporary Ileostomy and 2 trans appendiceal irrigation. 25 cases with BW< 1500 g (LBW) had M/F ratio 11/14, Mean B.W. 818 g, Mean G.A. 27 w, Caesarean section rate 70%, assisted ventilation 16/25. There were 8 inborn and 17 outborn. Gastrografin enema was successful in 6 out 8 inborn infants only, all referred within one week from birth. There were 12 perforations mainly among late referred LBW outborn.

**Conclusions:**

Meconium Related Ileus without Cystic Fibrosis responds to conservative management and softening enema in most of mature infants. In LBW clinical course is initially benign but as any long standing bowel obstruction management may present particular challenges. Clinical and plain radiographic criteria are reliable for making diagnosis and testing for Cystic Fibrosis may not be indicated. Enema may be resolutive when performed in a proper environment. Perforated cases may be confused with NEC which is excluded by clinical history, no signs of sepsis, lab signs missing, abdominal signs missing, typical radiological signs missing. The higher complication rate is recorded among cases delivered and initially managed in Neonatal Units without co-located Surgical Facilities. Early diagnosis and aggressive medical therapy may lead to higher success rate and help avoiding surgical interventions. Surgical therapy in uncomplicated cases, unresponsive to medical management, should be minimally aggressive.

## Background

Meconium abnormalities are at the origin of a series of neonatal intestinal obstructions, characterized by a wide spectrum of severity, from the benign meconium plug syndrome to the complicated meconium peritonitis. In 1999 Kubota [1] proposed the term Meconium Related Ileus (MRI) to comprehend different forms of meconium obstruction not associated to Cystic Fibrosis (CF) like Meconium Plug Syndrome (MPS) and Meconium Disease (MD). These relatively frequent and benign conditions need prompt recognition to exclude other forms of neonatal intestinal obstruction; among them MD is frequently associated to severe prematurity and low birth weight. The increasing survival rate of extremely preterm babies in the last twenty years, made MD to become an emerging and often challenging clinical problem. It results from combination of highly viscid meconium in the colon or terminal ileum and poor intestinal motility, resulting in mechanical bowel obstruction. CF and Hirschsprung's Disease (HD), when actively researched, are always excluded. Specific risk factors like maternal hypertension, maternal MgSO4 therapy, Caesarean delivery and maternal diabetes are believed to play a role in the pathogenesis of MD [2]. Clinical features consist of abdominal distension and bilious emesis following, in most of the cases, spontaneous meconium passage. Small bowel loops are typically distended on plain X-ray films, without fluid levels or pneumatosis [3]. There are not definitive criteria to manage MD; early diagnosed cases respond to conservative approach or meconium softening enemas. Delayed recognition leads to an increased risk of intestinal perforation, mainly among immature newborns, and often demands urgent surgery. This is a retrospective study about 55 infants, 25 of which were very low birth weight, presenting the clinical and radiological features of MD, managed in a single centre along a 16 years period.

## Methods

There were 55 infants referred in a period extending from 1992 to 2010 to the Pediatric Surgery Unit of the San Camillo-Forlanini Hospital. Thirteen patients were delivered at our Institutions (inborn - IB); all others were referred (outborn-OB) at median age of 6 days (range 1-30). Data from clinical charts and X ray studies were suggestive for a MRI. Management of our cases, included always Gastrografin enema, whenever overt perforation at referral was not present,. The contrast was diluted 50%with saline and dripped through an 8 F rectal tube from a 100 ml bottle hanging 50 cm above the patient, without any manual pressure. All enemas were performed in the Radiology suite, under fluoroscopy, without any complication. In most fragile LBW cases it was attempted first at bedside without fluoroscopy then, if unsuccessful, in the Operating Room under fluoroscopy. It was always essential for enema to reach the Ileum to improve the bowel obstruction. Elective surgery when elected after unsuccessful enema and persisting obstruction consisted, in the first years of our experience, in a temporary double barrel ileostomy. More recently we preferred a minimal Laparotomy, trans appendiceal bowel irrigation and Appendectomy. For perforated cases, peritoneal drain was employed only as a primary emergency tool, to reduce severe pneumo peritoneum, before formal surgical exploration.

Data collected from our series included:

- Obstetrical History

- Perinatal history and neonatal clinical data

- Place of birth (inborn or outborn) and time before referral

- Clinical course and complications

- Radiological features

- Management

- Outcome

Data about Very Low Birth Weight (LBW < 1500 g.) were considered separately and compared to those from patient's ≥ 1500 g.

## Results

Males-Females ratio was 27/28. All clinical histories were characterized by:

- No spontaneous passage of meconium

- Distended abdomen and bilious emesis on referral

- Oral feeding not started

- Necrotizing Enterocolitis (NEC) excluded by radiological or surgical criteria

- HD and CF excluded by rectal biopsy ad genetic probe before discharge

Mean gestational age was 33,05 weeks (range 24-40) and mean birth weight was 2051 (range 500-4000). Prenatal diagnosis of suspected intestinal abnormality was never recorded. Twenty-five infants in our series (45%) were LBW versus 30 with a BW ≥1500 g (Table 1); 8 of them were IB and 17 OB. Mean Apgar index among LBW at 1' was 5.3; sixteen patients needed mechanical ventilation for important co morbidities typically associated with prematurity, included respiratory distress syndrome.

**Table 1:** Characteristics of 55 infants with MRI observed between 1992 and 2010

	BW ≥ 1500 g	BW< 1500 g (LBW)	p
N.of Cases	*30 *	*25 *	

M/F ratio	*16/14*	*11/14*	*n.s*

Mean B.W.	*3052 g (range 1660-4000)*	*818 g (range 500-1480)*	*< 0.0001*

Mean G.A.	*37 w (range 32-40)*	*27 w (range 24-31)*	*< 0.0001*

Cesarean sections rate	*40%*	*70%*	*n.s*.

Maternal Diabetes	*6,6%*	*8%*	*n.s*

Maternal Hypertension	*18%*	*11%*	*n.s*.

Mean Apgar index at 1'	*8*	*5.3*	*< 0.005*

Assisted Ventilation	*2*	*16*	*< 0.0001*

Table 2 reports differences in clinical course and management between immature infants with MRI and others. Among our series of patients, 32 out of 43 (74%) bowel obstructions with no signs of perforation or peritonitis responded to Gastrografin enema. Surgery was very uncommon (10%) among patients ≥ 1500 g of BW who almost always responded to softening enema. Among LBW only 7 infants out of 25 (28%) passed some smears of meconium before developing a significant abdominal distension and bilious emesis, unresponsive to rectal stimulation and water-soluble enema. Among the 8 IB, LBW patients, only one patient had an isolated intestinal perforation successfully managed by primary resection. Two had an elective surgery (Transappendiceal Irrigation), included one patient who had a previous unsuccessful enema. Gastrografin enema succeeded resolving obstruction in 5 out of 8 IB cases. The 17 OB LBW infants were the more clinically compromised group. They were usually late referred with an overt or pending perforation (64%) after a long lasting unresolved obstruction and all required surgery. A central venous line was positioned in 19 out 20 operated LBW cases. No sign of NEC was found at exploration. Among surgical options adopted for LBW infants with complicated MRI, trans appendiceal bowel irrigation was successfully performed in 8 who did not respond to Gastrografin enema, while 1 of 2 perforated cases who had primary resection and 7 of 10 managed by temporary stoma died within a week from operation for severe sepsis (5) and intracranial haemorrhage (3). Table 3 reports impact of patient's characteristics on management and outcome. The higher mortality rate (58%) was observed among the 17 LBW Outborn patients. Survival was significantly influenced by GA, BW, prenatal maternal risk factors and secondarily by Cesarean section rate and place of delivery. Softening enema showed a lower success rate among low weight, premature infants.

**Table 2:** Clinical course and management of 55 infants with Meconium Related Ileus

	30 - BW ≥ 1500 g		25 - BW< 1500 g (LBW)	
**Inborn/Outborn**	5/25		8/17	

**Feeding before onset**	None		None	

**Mean time of referral for OB**	2,4 Days (1-7)		11 Days (7-29)	

**Clinical features**	10 meconium plug Syndrome 4 small left colon Syndrome 16 meconium ileus without CF		25 meconium ileus without CF *perforated: - 11 among OB* *- 1 among IB*	

**Management**	**IB** Enema 5	**OB** Enema 22 Ileostomy 1 Transappendiceal Irrigation 2	**IB** Enema 5 Resection 1 Transappendiceal Irrigation 2	**OB** Resection 1 Ileostomy 10 Transappendiceal Irrigation 6

**Table 3:** Impact of patients' characteristics on management and outcome

	Responding to Enema	Requiring Surgery	*p*	Survived	Dead	*p*
**No of cases**	32	23		45	10	

**Mean BW (g)**	2699	1171	*< 0.0001*	2360	830	*< 0.0001*

**Mean GA (w)**	36	28	*< 0.0001*	26.6	34,6	*< 0.0001*

**CS (cases)**	17	12	*ns*	20	9	*0.012*

**Prenatal Maternal Risk Factors (cases)**	5	8	*ns*	4	9	*< 0.0001*

**Inborn/Outborn**	10/22	3/20	*n.s*.	13/32	0/10	*< 0.05*

## Discussion

There is a wide spectrum of neonatal conditions having in common an intestinal obstruction by ispissated endoluminal content, in absence of mechanical obstacle, CF or HD. Rickman and Boeckman, in 1965, first reported a neonatal meconium obstruction in absence of CF and Vinograd et al, in 1983, reported the first series of this syndrome in LBW. Kubota at al [1]. were the first to propose the term "Meconium Related Ileus" MRI to cover the so called Meconium Plug Syndrome, Small Left Colon Syndrome and Meconium Disease, in absence of CF and HD, concluding that these conditions were essentially the same entity with a different degree of severity. All were characterized by a combination of highly viscid meconium and poor intestinal motility, low grade obstruction, benign systemic and abdominal examination, distended loops without air fluid levels. Added risk factors are severe prematurity and low birth weight, Caesarean delivery, maternal therapy with MgSO4 [2]. MRI in the term infant is often characterized by lack of meconium passage in the first 24-48 hours responding to a simple conservative treatment based on rectal stimulation and irrigation [4]. In our series, all infants ≥1500 g BW were seen within the fist week of life and responded to conservative management and Gastrografin enema in 90% of the cases. Unlike their mature counterpart, LBW babies presented first clinical sign usually around the second week of life, although the condition can present at any time after birth [2]. These infants, who often passed some smears of meconium, have a typically progressive abdominal distension with palpable bowel loops and no sign of peritonitis. Plain abdominal film shows distended small bowel loops without air fluid levels or pneumatosis. These findings are enough to make diagnosis and exclude other forms of intestinal obstruction, mainly NEC. Testing for Cystic Fibrosis may not be indicated. Once the obstruction occurred, the risk of perforation becomes higher and is estimated around 30%. Conservative management can be successfully applied in two thirds [4]. Nevertheless, diagnosis and proper management of MRI among LBW continue to be delayed and many infants are referred already perforated as occurred in 65% of our OB, seen by the surgeon some days after obstructive symptoms appeared. This rarely occurred among IB (12,5%). Place of birth was one of the secondary factors affecting survival in our series; mortality remains strongly related to GA, BW and complicated pregnancy.

No definitive management of MRI has been stated until now, mainly for LBW cases [5]. Since Noblett introduced Gastrografin enemas to treat meconium ileus [6], this became the mainly accepted conservative treatment even for MRI [1,3,7,8] despite its efficacy is not yet universally recognised [9]. Success rate is estimated around 80% and is strictly time dependent; failure is reported among cases after 14 days from diagnosis [5]. Recently Iopamidol has been preferred to Gastrografin by some Authors [10] for its lower osmotic pressure, with the same rate of success. In a recent multi institutional review [11] a decreasing use of softening enema for MRI has been documented along the last decade. An earlier surgical approach is preferred to enema especially when it requires multiple attempts. The reason for this change may lie in the introduction of new osmotic agents, less effective of Gastrografin, or in the reluctance of Radiologist to repeat enema in most critical cases. In our series lox BW and GA were associated to a most frequent recourse to surgery. This certainly can be explained by the higher rate of bowel perforations at referral among our LBW infants but it could be also attributed to a propensity to refrain from using repeated cleansing enema in most fragile cases, especially when late referred.

Where enema has to be performed, especially when dealing with fragile LBW infants, is still matter for discussion [4,5]. Transport to Radiology suite is sometimes difficult to arrange for artificially ventilated patients and fluoroscopy guidance is not possible in the Neonatal ICU [3,8]. Fluoroscopy is essential to document contrast medium passing ileocecal valve and mixing with intestinal content, to get an effective clinical result. An alternative could be to perform the enema in Operating Room, when available, which offers the advantage to face promptly perforative complications [5] This method must be preferred in most critical patients who did not respond to bedside enema. Warming of radiology suite and proper assistance to these neonates must be guaranteed during all the procedure when not done in the Operating Room. Surgery was decided on elective basis in 11 cases, after unsuccessful enema, and on emergency basis in 12 for an overt perforation.

Instillation of *N*-Acetylcysteine via nasogastric tube has been reported to decreases stool viscosity by 99% in 6 hrs in early diagnosed cases [8] but was never attempted among our patients. Recourse to surgery is frequently reserved to late referred cases that present a perforation rate of 28% [2]. Whenever MRI leads to intestinal perforation prompt abdominal exploration is mandatory. Percutaneous drain in extremely premature infants has been suggested but its real role has not yet been established [12]. Elective surgical options for MRI vary from enterotomy, irrigation and primary closure to temporary stoma. Although this technique in commonly used in full term infants, irrigation may be dangerous in LBW infants and iatrogenic bowel injury during this manoeuvre has been reported [8]. When necrotic bowel is present, minimal resection and enterostomy is recommended.

## Conclusions

MRI in absence of CF is a relatively uncommon neonatal condition, which affects mainly LBW that showed an increasing occurrence in the last twenty years as a result of increasing survival rate of this fragile group of patients. Prompt recognition associated to an early and "aggressive" conservative management is essential to prevent complications and spare surgical interventions. Gastrografin enema is proved to be safe and an effective if promptly implemented in a suitable environment and by experienced hands. It is essential to administrate the enema under fluoroscopy to document passage of contrast medium through ileo cecal valve. Only few cases with severe ileal meconium impaction may require enterotomy, irrigation and primary closure. Perforation is frequently reported among LBW and seems to be correlated to delay in diagnosing and referring to surgical attention. Simple surgical measures must be recommended in these cases. Limited resection and temporary stoma seem to be the most effective procedure as percutaneous peritoneal drain has been demonstrated to be only of temporary help to resolve pneumo peritoneum and secondary respiratory distress [13].

## Competing interests

The authors declare that they have no competing interests.

## Authors' contributions

PFV and CR had a primary role in study design, data analysis and data interpretation.

The preliminary proof of the paper was written internally by the company and deeply revised by BV and LO.

Company had the right to revise the version to be submitted but the final decision about paper submission relied entirely on AC. AC also revised critically the paper version to be submitted.

All authors have read and agreed to its content.
